# Optimized Varian aSi portal dosimetry: development of datasets for collective use

**DOI:** 10.1120/jacmp.v14i6.4286

**Published:** 2013-11-04

**Authors:** Ann Van Esch, Dominique P. Huyskens, Lukas Hirschi, Stefan Scheib, Christof Baltes

**Affiliations:** ^1^ 7Sigma QA‐team in Radiotherapy Physics Tildonk Belgium; ^2^ Radiotherapy Association Ste. Elisabeth Namur ‐ Centre Hospitalier Mouscron Namur and Mouscron Belgium; ^3^ Department of Nuclear Medicine and Radiotherapy Triemli Hospital Zurich Switzerland; ^4^ iLab Varian Medical Systems Baden Switzerland

**Keywords:** portal dosimetry, preconfigured datasets

## Abstract

Although much literature has been devoted to portal dosimetry with the Varian amorphous silicon (aSi) portal imager, the majority of the described methods are not routinely adopted because implementation procedures are cumbersome and not within easy reach of most radiotherapy centers. To make improved portal dosimetry solutions more generally available, we have investigated the possibility of converting optimized configurations into ready‐to‐use standardized datasets. Firstly, for all commonly used photon energies (6, 10, 15, 18, and 20 MV), basic beam data acquired on 20 aSi panels were used to assess the interpanel reproducibility. Secondly, a standardized portal dose image prediction (PDIP) algorithm configuration was created for every energy, using a three‐step process to optimize the aSi dose response function and profile correction files for the dosimetric calibration of the imager panel. An approximate correction of the backscatter of the Exact arm was also incorporated. Thirdly, a set of validation fields was assembled to assess the accuracy of the standardized configuration. Variations in the basic beam data measured on different aSi panels very rarely exceeded 2% (2 mm) and are of the same order of magnitude as variations between different Clinacs when measuring in reference conditions in water. All studied aSi panels can hence be regarded as nearly identical. Standardized datasets were successfully created and implemented. The test package proved useful in highlighting possible problems and illustrating remaining limitations, but also in demonstrating the good overall results (95% pass rate for 3%,3 mm) that can be obtained. The dosimetric behavior of all tested aSi panels was found to be nearly identical for all tested energies. The approach of using standardized datasets was then successfully tested through the creation and evaluation of PDIP preconfigured datasets that can be used within the Varian portal dosimetry solution.

PACS number: 87.55.km, 87.55.Qr, 87.56.N_

## I. INTRODUCTION

With the rise of intensity‐modulated treatment techniques — be they static gantry IMRT or VMAT — came the need for thorough, yet efficient, patient QA. The portal imager is a much appreciated measurement device for routine use because of its prompt setup, its easy data acquisition, and its high resolution. An extensive literature overview on the research devoted to the portal imaging panels for radiotherapy dosimetry was written by Van Elmpt et al.^(1)^

Although different portal imaging devices exist, a commonly used model, and the one discussed in this study, is the Varian amorphous silicon (aSi) panel. In order to be able to perform portal dosimetry pretreatment patient QA, one needs to have both a measured and expected dosimetric image. This is not as straightforward as one might hope because the aSi panels are far from water‐equivalent. Numerous studies have shown that they display a dosimetric response that is quite different from the behavior observed in a water phantom, even when measuring at the same depth.^2^, ^3^, ^4^, ^5^, ^6^, ^7^

Different commercial solutions are available to attend to this issue. Some try to convert the measured aSi image to dose to water at a certain depth and subsequently compare it to the dose calculated by the TPS in a water‐equivalent phantom.^(8)^ An important advantage of this approach lies in the fact that the algorithm used for the patient dose calculation is also used for the portal dose image calculation (albeit in simplified phantom conditions and at only one depth). As a drawback, the aSi dosimetric image needs to be processed considerably in order to be converted into dose to water. The use of a direct, rather than indirect, aSi detection system could provide an elegant answer to this problem,^(9)^ but such solutions are not commercially available yet and have the major drawback that they lead to poor image quality.^(^ ^10^ ^,^ ^11^ ^)^ Other approaches leave the measurement untouched, but rely on a separate beam model to derive the expected portal dose image.^(^ ^6^ ^,^ ^12^, ^13^, ^14^ ^)^ The Varian portal dosimetry follows this approach: it does not use the patient dose calculation algorithm but relies on the portal dose image prediction (PDIP) algorithm to calculate the expected aSi dose image based on the theoretical TPS photon intensity matrix, the main collimator positions, and the total monitor units (MUs). It is a highly efficient solution and very sensitive to error detection in the actual fluence calculation or MLC delivery.

Unfortunately, when implementing the algorithm according to the official procedure, results can be suboptimal, giving rise to false error reports, as documented by, for example, Vinall et al.^(15)^ The main reason for this is not the prediction algorithm itself, but the chosen method for the dosimetric calibration of the imager panel — more precisely, the profile correction used during the calibration of the imager panel. The dosimetric calibration of the imager panel consists of the acquisition of a ‘dark’ and ‘flood’ field. The dark field serves to correct for the background signal when no radiation is present. The flood field is a very approximate method to correct for variations in pixel sensitivities by simply normalizing the response of all pixels to a large (typically $40\times 30\;{\text{cm}}^{2}$) open field acquisition. This correction was originally developed for imaging purposes only, for which it is of no importance that it washes out all variations (off‐axis beam intensity, field penumbra, or spectrum related) in the beam at off‐axis distance. For dosimetric purposes, however, this calibration method is not sufficient as it will result in a perfectly flat dosimetric image of a $40\times 30\;{\text{cm}}^{2}$ open field, even in the penumbra region. To rectify this, a profile correction file can be provided during the subsequent dosimetric calibration. Although the original PDIP algorithm was developed using a 2D profile correction based on a film measurement at 8 mm depth^(6)^ (using the same field size as used during the flood‐field calibration), this approach was never implemented in the official release as film measurements are too cumbersome to be universally applied in a reliable way. The commercially adopted approach — using the diagonal profile of a $40\times 40\;{\text{cm}}^{2}$ field measured in a water phantom at $d_{\text{max}}$ instead — may be more reproducible and yields adequate results in the central part of the detector panel. But it is considerably less accurate outside of this central area as the diagonal profile is not acquired at the same water‐equivalent depth and the penumbra of the delivered rectangular flood field is not correctly accounted for. Deviations between measured and predicted dose reach 2% at 10 cm off‐axis for 6 MV and increase further towards the edges of the detector panel.^(16)^

Instead of using the official diagonal profile, Bailey et al.^(17)^ use a one‐dimensional beam profile correction that is extracted from the prediction of a $40\times 40\;{\text{cm}}^{2}$ open field, considerably improving the off‐axis agreement between measured and calculated images. Vinall et al.^(15)^ take it one step further and introduce a two‐dimensional profile correction matrix, generated from the image prediction of a $40\times 30\;{\text{cm}}^{2}$ rectangular open field (i.e., the field size used during flood‐field calibration). In addition to the suboptimal profile correction, the presence of inhomogeneously backscattered dose from the imager arm during the flood‐field calibration is disregarded in the commercial solution. The impact of the backscatter of the EPID arm has been reported in literature and is known to cause local deviations of up to 2%‐3%, depending on the used clinical field size and beam energy. Some studies have added a lead shielding to the back of the imager panel to provide a more uniform backscatter.^18^, ^19^, ^20^ The research groups of Rowshanfarzad et al.^(21)^ and Greer et al.^(22)^ have published a number of studies and a possible solution to the backscatter issue They have performed off‐arm measurements to better quantify the backscatter contribution and to obtain a backscatter‐free, flood‐field image. By adding a backscattered dose prediction (using a backscatter kernel) to an existing EPID dose prediction model, they aim to model the backscatter in the predicted image. This EPID dose prediction model was based on the energy fluence model in the Pinnacle TPS. Within the Eclipse (Varian Medical Systems) environment, it is currently not possible to add an inhomogeneous backscatter component to the predicted images. As an alternative to physically removing the cassette from its arm, Vinall et al.^(15)^ deduce the impact of the backscatter that was present during the flood‐field calibration from the asymmetries observed in a series of subsequent open field measurements. They then include the estimated backscatter into their above‐mentioned 2D profile correction matrix, after which it is applied to all future dosimetric image acquisitions. Both approaches offer a simplified, field size independent solution that improves results for small‐to‐moderate field sizes at the cost of large field accuracy. A more sophisticated field size‐specific backscatter correction algorithm was proposed by Berry et al.^(23)^

In spite of the fact that solutions for the field profile and backscatter corrections have been developed and tested, they often require considerable measurements and numerous image manipulations and/or transformations and are, therefore, too cumbersome to implement in most radiotherapy departments. The goal of this work is to make standardized preconfigured datasets, including the improvements reported by Vinall et al., for the different photon energies and aSi imager panels available on the Varian Clinac and to subject the developed datasets to multicentric testing. We have opted for the solution published by Vinall et al.^(15)^ because it combines both a 2D profile correction with an (albeit approximate) backscatter correction and has the major advantage that, once configured, it can be used entirely within the Eclipse environment without the need for further image transfer and/or additional image processing.

## I. MATERIALS AND METHODS

### A. Intercomparison of the dosimetric characteristics of various aSi panels

The use of a preconfigured portal dosimetry package can only be justified for aSi panels that display near‐identical dosimetric behavior. This is, therefore, a mandatory prerequisite to be investigated first. To study the interpanel variations, data gathered on 20 different aSi panels (Varian Medical Systems, Palo Alto, CA) for the most commonly used beam energies were compared (10 panels for 6 MV, five panels for 10, 15, 18, and 20 MV each). All these panels were mounted on Exact arms on Varian dual energy Clinacs (Clinac 21EX, 23EX, 2300IX, 2100C/D). The age of the panels varied from brand new to a few years of service in clinical routine. Frame readout was performed with the IAS3 image acquisition system in full (aS1000) or half resolution (aS500), depending on the available licenses. The dosimetric characteristics of the Clinacs (output factors, depth dose, and beam profile measurements) were also intercompared to avoid misinterpretation of possible variations in the aSi measurements that should really be attributed to the Clinac instead. The variability of different aSi panels is assessed through field size dependence, dose linearity measurements, characteristics of the backscatter of the Exact arm, and the aSi detector response function.

For the panel intercomparison, the portal imagers were calibrated in dosimetric acquisition mode at isocenter according to the official portal dosimetry calibration procedure, applying a dark and flood‐field (by means of a $40\times 32\;{\text{cm}}^{2}$ field irradiation) correction, an absolute calibration with a $10\times 10\;{\text{cm}}^{2}$ field, defining 100 MU to correspond to 1 aSi calibrated unit (CU), and a beam profile correction. However, instead of using the diagonal profiles measured in a water phantom at $d_{\text{max}}$, we applied the same, perfectly flat profile correction to all panels to allow easy intercomparison, temporarily removing the impact of possible differences in beam profiles between different machines.

#### A.1 Field size dependence

Output factors were acquired with the imager panel at isocenter for field sizes ranging from 2×2 to $40\times 30\;{\text{cm}}^{2}$.

#### A.2 Linearity

The linearity of the detector response as a function of monitor units was investigated by delivering a $10\times 10\;{\text{cm}}^{2}$ field with MU ranging from 10 to 1000. The linearity was investigated for all energies, for clinical dose rates from 100 to 600 MU/min, and for both acquisition resolution modes.

#### A.3 Backscatter of the Exact arm

The tests performed during this study have a double purpose. Firstly, we aim to verify the reproducibility over different imager panels and Exact arms. Secondly, we aim to quantify the effect for all available energies in order to derive a backscatter correction similar to the one reported by Vinall et al.^(15)^ For both purposes, dosimetric images were acquired for a series of static open fields with X=40cm and Y ranging from 2 to 32 cm in steps of 1 cm for every energy. The magnitude and location of the backscatter can be estimated on the basis of the rectangular field measurement series, more precisely on asymmetries observed in the longitudinal field profiles. Without the backscatter, these field profiles would be perfectly symmetric.

#### A.4 aSi detector response function

The aSi detector response function describes the photon scatter within the detector. Although the dose spread characteristics of the aSi panel are inherently reflected in the output factor behavior, an additional test image was acquired and intercompared for the different panels. The test pattern used for this is based on the same principle as the dynamic pyramid‐shaped test image^(6)^ used for the official algorithm configuration: using a fixed main collimator opening, the dose measured in the center of different MLC openings is primarily dependent on the photon scatter within the aSi material. (The off‐axis fluence variation of the treatment beam is corrected out through the ‘flat’ profile correction.) In contrast to the official configuration procedure, the test uses a static MLC‐defined field rather than dynamic sweeping gap MLC to minimize the effect of differences in MLC characteristics (dosimetric leaf gap and MLC transmission) within the irradiated area.

### B. The making of standardized configurations

The Eclipse portal dose image prediction (PDIP) algorithm in the current Varian Portal Dosimetry solution convolves the theoretical TPS photon intensity matrix with a detector response function, referred to as the aSi single pencil beam kernel. This theoretical photon intensity matrix is the product of the ‘actual’ fluence and a beam intensity profile; the ‘actual’ fluence is the beam‐open fractional MU matrix as calculated from the MLC leaf motions and the beam intensity profile takes the off‐axis variation of the photon fluence in an open beam into account. The latter is a simple diagonal profile calculated by the TPS beam configuration software from basic measurements in water at depth of dose maximum. The absolute level of the predicted image is achieved by also applying a collimator scatter factor derived from the output factor measurements and the calculated phantom scatter factors. For a detailed description of the PDIP algorithm, we refer to Van Esch et al.^(6)^

The configuration of the PDIP algorithm requires output factor measurements performed with the aSi panel, the beam intensity profile and a set of pyramid‐shaped test images from which the algorithm configuration derives the aSi pencil beam kernel as the weighted sum of 9 Gaussian functions with predefined widths (ranging from 0.8 to 400 mm). The relative weights of the different Gaussian contributions are optimized during the algorithm configuration.

During the dosimetric calibration of the imager panel, the software calls for a profile correction file to account for the actual beam profile of the large open rectangular field used during the flood‐field calibration.

For the creation of the standardized configuration we therefore proceeded as follows:

1) Output factor tables. For the output factors, mean output factor tables were generated for all available energies from the sets of beam data used during the intercomparison of the different panels.
2) Beam intensity profile. For the beam intensity profile, we first intercompared the intensity profiles of the different treatment machines used in this study. These were derived from previously acquired diagonal beam profiles at the depth of dose maximum in a water phantom by the local physicists (using a variety of water phantoms and detectors, depending on the local equipment) for the configuration of the Eclipse photon dose calculation algorithm (be it the analytical anisotropic algorithm (AAA) or the Pencil beam convolution (PBC) photon dose calculation algorithm). From this dataset, a final, mean intensity profile was extracted to provide the best possible match with all treatment machines.
3) Beam profile correction. For the creation of the beam profile correction files and the aSi scatter kernels, we applied the three‐step process reported by Vinall et al.^(15)^

In a first round, for every energy we converted diagonal beam profile measurements $\left(40\times 40\;{\text{cm}}^{2}\right)$ at $d_{\text{max}}$ into the official 1D profile correction file format. Although this profile correction procedure is known to result in increasingly large deviations between prediction and measurement towards the edges of the detector, it provides adequate results in the central part of the detector plane. As the pyramid‐shaped test image is located within this central part, the use of this official profile correction suffices for an initial algorithm configuration.

In a second step, a $40\times 32\;{\text{cm}}^{2}$ open field portal dose image is calculated by means of this initial algorithm configuration. The predicted image is converted into a 2D profile correction — it is resampled to map the imager pixels and reformatted to allow its use in the current portal dose calibration software.

Thirdly, we have incorporated the backscatter of the Exact arm into this 2D profile correction image by pixelwise multiplying it with the effect of the backscatter as estimated from the rectangular open field measurement series.^(15)^ As such, a 2D beam profile correction matrix is obtained accounting for the true field size during the flood‐field calibration and introducing an approximate backscatter correction.

4) Single pencil beam kernel. Because of the above beam profile correction procedure, the PDIP single pencil beam kernel was also derived in two steps. An initial kernel was derived by means of the pyramid‐shaped test images acquired with the official profile correction calibration procedure. Once the 2D profile correction was available, the portal imager was recalibrated and a new test image was acquired. The PDIP single pencil beam kernel was then fine‐tuned by importing the test image with the improved profile correction into the PDIP basic beam data and rerunning the algorithm configuration.

Although in theory, the above procedure should be iterated until convergence, the fine tuned kernels do not differ enough from the initial kernels to result in noticeable changes in the shape of the large open field prediction and no iterations were, therefore, performed in practice.

As such, a standardized configuration package with corresponding 2D profile correction matrices was generated for all energies.

### C. Multicentric testing of the standardized configurations

The above‐generated standardized configurations were tested on‐site in seven different radiotherapy departments (on 12 Clinacs in total). The preconfigured beam data for the PDIP algorithm were simply imported into the Eclipse treatment planning software, while the portal imager was calibrated in dosimetric mode using the corresponding 2D profile correction file.

A dedicated set of validation fields was then imported, the actual fluences were recalculated with the local MLC parameters, and the portal dose images were subsequently predicted, measured, and analyzed. A selection of ten static fields with varying field sizes (from 3×3 to $40\times 30\;{\text{cm}}^{2}$) verify both the output factor behavior and the correct application of the profile correction, all delivered with 100 MU to exclude inaccuracies that could arise from linearity issues. The linearity is separately addressed by delivery of a $10\times 10\;{\text{cm}}^{2}$ field with 20, 50, 100, 200, and 500 MUs.

In addition, a number of dynamic IMRT fields were chosen. Some artificial patterns were applied to assess the precision of the preconfigured portal dosimetry package under extreme circumstances. Some clinical IMRT fields (prostate, head and neck, and breast compensator) were also included to look into more clinically relevant cases. Depending on the available licenses, large IMRT fields with multiple MLC carriage positions were either split into two individual IMRT fields or treated as a single large IMRT field for which the main collimators and MLC carriage move during an automatic beam hold. In the former case, the two separate images of the split IMRT field were combined into a single composite image prior to evaluation.

Similar to the IMRT PDIP validation, test plans for RapidArc (Varian Medical Systems) portal dosimetry included RA fields generated on artificial structures (a central and off‐axis cylinder^(24)^), as well as two clinical cases (prostate, head and neck). No correction algorithm for the imager sag as a function of gantry angle was applied, as this is not possible when using the integrated image acquisition mode (for which no separate image information is stored as a function of gantry angle).

## II. RESULTS

### A. Intercomparison of the dosimetric characteristics of various Varian aSi panels

#### A.1 Field size dependence

**Figure 1:**
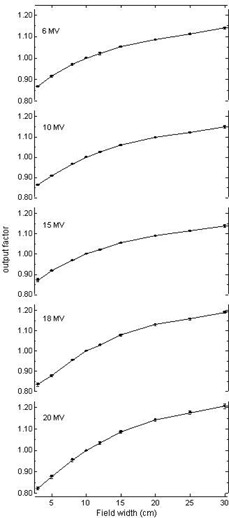
Output factors measurements averaged over all tested aSi panels. Error bars indicate the maximum observed deviation from the mean value.

As can be seen from Fig. 1, output factors measured on different aSi panels are identical within 1% for almost all clinically used field sizes and for all energies. The figure displays the mean output factors, and the error bars indicate the maximum observed deviation from the mean value. Standard deviations are too small to be displayed on the graph as they range between 0. 11% and 0.27% for the different datasets. Differences of maximum 2% are only measured for field sizes beyond $30\times 30\;{\text{cm}}^{2}$ or below $3\times 3\;{\text{cm}}^{2}$. The observed differences are of the same order of magnitude as the output factor variations between different Clinacs when measuring in reference conditions in water. (The near‐identical behavior of output factors among different Clinacs is to be expected due to the almost identical design of the Clinac heads, and has been confirmed in literature^(25)^). It can therefore be concluded that, with respect to field size dependence, all studied aSi panels can be regarded as nearly identical.

#### A.2 Beam intensity profile

From the intensity profile evaluation, we also observe that intermachine comparisons entirely pass gamma criteria of 2%,2 mm, as is again to be expected from the identical Clinac head design.

#### A.3 Linearity

**Figure 2:**
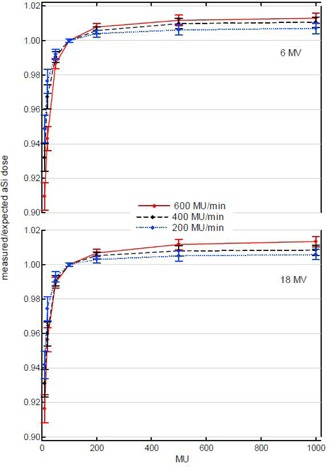
The mean ratio of the measured to theoretically expected integrated dose value in calibrated units (CU) as a function of MU for a $10\times 10\;{\text{cm}}^{2}$ field. Results of the linearity tests are shown for different dose rates for low (6 MV) and high (18 MV) energy. All dose rates were calibrated to yield 1 CU per 100 MU. Error bars indicate the maximum observed deviations from the mean value.

The aSi acquisition mode on the IAS3 acquisition system exhibits deviations from a perfectly linear dose response. Figure 2 displays deviations up to 10% from the theoretically expected value for dose acquisitions of 10 MU, whereas dosimetric images of 1000 MU can be off by 1% to 2%. All deviations are relative to the 100 MU deliveries, as these represent the reference conditions for the absolute dosimetric calibration of the acquisition mode. Figure 2 displays the deviation per dose rate, averaged over all panels. The observed deviations are not perfectly reproducible between all aSi panels, but do show a very similar tendency; error bars indicate the maximum deviation from the average value. For all panels and all energies the largest deviations are observed for images encompassing only a small amount of frame acquisitions (i.e., lowest MU and highest dose rate). For the more clinically relevant MU (between 50 and 500 MU) deviations remain within 1% for all dose rates and all energies. Ion chamber measurements in water show near‐perfect linear behavior over the whole range of tested MU, proving that the observed nonlinearity is related to the image acquisition rather than to the machine performance. This nonlinearity of the integrated acquisition mode is in contrast to earlier findings, reporting very linear behavior down to only a few MU.^(^ ^6^ ^,^ ^26^ ^)^ Detailed investigation of the acquisition mode parameters confirm this; it was found that the current integrated acquisition mode is missing the partial frame at the very end of the acquisition. After beam‐off, apart from finishing the frame that is in the process of being read out, the acquisition mode should read one more frame to make sure all deposited dose is included in the final image. Although this additional frame read‐out was programmed in earlier software versions,^(^ ^6^ ^,^ ^26^ ^)^ in the current version of the IAS3 software, this appears not to be so anymore, thereby explaining the observed behavior as a function of total frame acquisitions. In a worst‐case scenario, the resulting impact on the integrated acquisition was estimated to be 0.5 MU.

#### A.4 Backscatter of the Exact arm

**Figure 3:**
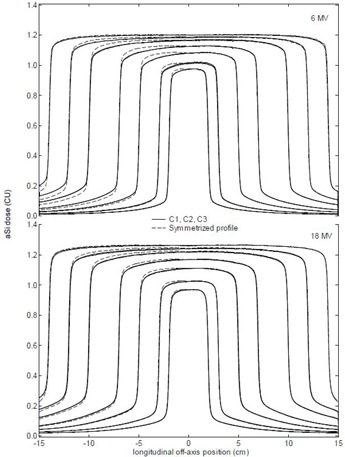
Longitudinal image profiles showing the impact of the backscatter of the Exact arm on the open field acquisitions for different field sizes (X=40cmandY=4,6,10,14,20,24,28cm) for low (6 MV) and high (18 MV) energies. The figure displays data for three randomly selected imager panels (C1, C2, and C3) per energy only, using the same solid line type for all imagers, as they superimpose beyond distinction. As a visual aid, the symmetric profiles (dashed lines) were generated by mirroring the half profiles acquired on the lower half of the panel (corresponding to the right half of the profiles on the graph) where the impact from the inhomogeneous backscatter of the arm is minimal.

The impact of the backscatter of the Exact arm during the $40\times 32\;{\text{cm}}^{2}$ flood‐field calibration is clearly visible in the rectangular open field measurements shown in Fig. 3. The flood‐field image contains a maximal backscatter component. As the collimator opening is reduced, so is the backscatter contribution. This is especially visible in the inplane direction in the ‘upper’ half of the imager panel (i.e., the part that is closest to the gantry) where the arm mount and the metal support bar provide the largest sources of backscatter. While the backscatter component reduces with field size, the flood‐field image still corrects for maximal backscatter and the final dose is reported to be lower than it actually is. The ‘lower’ half of the imager panel (i.e., the part furthest from the gantry) suffers considerably less from the inhomogeneous backscatter effect of the arm. The symmetric field profiles displayed in the figure are obtained by mirroring the half profile from the lower part of the imager panel to aid the visualization of the asymmetry between both sides. The open field measurements of the different panels superimpose to the point that the different profiles can hardly be distinguished from one another. For clarity, Fig. 3 shows data for a (random) selection of three imager panels, but the backscatter effect is nearly identical for all tested Exact arms and imager panels. As previously reported in the literature,^(^ ^15^ ^,^ ^18^, ^19^, ^20^, ^21^, ^22^, ^23^ ^)^ it can clearly be observed that the effect is most significant for the lowest energies, destroying the symmetry of the longitudinal field profiles for fields with Y<~25cm. Most unfortunately, maximum deviations (~3%) are observed for the very commonly used clinical field dimension Y=10to15cm.

#### A.5 aSi detector response function

**Figure 4:**
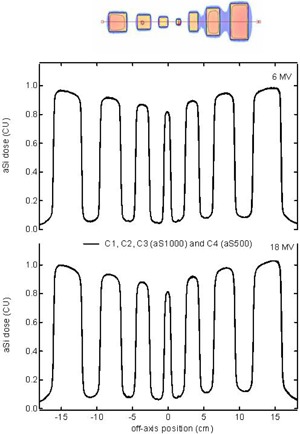
Static MLC test image assessing the reproducibility of the photon scatter characteristics of different aSi panels (C1, C2, C3 (aS1000) and C4 (aS500)). A representative selection of four different datasets is shown per energy. All are displayed as solid lines as they superpose to the point where they can hardly be distinguished from each other in the graph.

In agreement with the near‐identical output factor behavior, the very similar scatter characteristics of the different panels are confirmed by the static MLC test image. Figure 4 shows a selection of typical horizontal line profiles for a number of imager panels for different energies. Even between aS500 and aS1000 acquisitions, all differences are insignificant and within the noise level of the image acquisition. This single field test adds justification to the use of a single aSi detector response function per energy for all imager panels.

### B. Distillation of standardized configurations

From the panel intercomparison results, the mean output factor tables and mean intensity profiles were selected for use in the standardized configuration sets.

**Figure 5:**
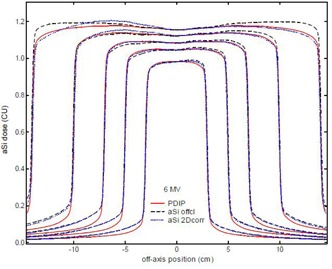
Open field longitudinal profile (6 MV) comparisons showing the predicted profile (solid line) vs. the measured profile with the official profile correction (dashed line) and the 2D profile correction including the backscatter (dotted line).

The three‐step process for the creation of the aSi pencil beam kernels and the corresponding 2D profile correction files resulted in final kernels for all energies, as shown in Table 1, and considerably improved the agreement between predicted (solid lines) and measured open field profiles, as shown in Fig. 5. The increasingly poor agreement towards the edges of the detector — typically observed when using the official diagonal profile correction (dashed lines) — exists no longer as a result from the two‐dimensional correction approach (dotted lines). The incorporated backscatter correction considerably improves the symmetry of the longitudinal open field profiles for clinical field dimensions up to 15 cm. As a tradeoff, the backscatter contribution appears in the aSi image acquisition of the larger open fields (introducing a maximum discrepancy of 1% to 3% for Y going from 20 to 30 cm), but overall agreement is still considerably better than with the official diagonal profile correction.

**Table 1:** Relative weights of the different Gaussian contributions for the aSi pencil beam kernels for all energies. The corresponding Gaussian widths are predefined and listed in the first column

*Gaussian Width*	*6 MV*	*10 MV*	*15 MV*	*18 MV*	*20 MV*
0.80	0.4226	0.3854	0.3026	0.2548	0.2840
1.74	0.1828	0.1763	0.2102	0.2047	0.1750
3.78	0.0210	0.0197	0.0890	0.0894	0.0595
8.23	0.0572	0.0498	0.0396	0.0320	0.0404
17.90	0.0446	0.0775	0.0326	0.0653	0.0736
38.90	0.0197	0.0131	0.0444	0.0244	0.0474
84.6	0.0701	0.0685	0.0726	0.0992	0.0874
184.00	0.0889	0.1026	0.0996	0.1157	0.1143
400.00	0.0929	0.1072	0.1084	0.1145	0.1184

### C. Multicentric testing of the standardized configurations

The on‐site import of the preconfigured beam data and the corresponding 2D profile correction file in the test centers proved to be very easy, allowing setup of the complete portal dosimetry solution in less than one hour. Although the system could in theory then immediately be used for routine verification of clinical fields, running the predefined set of validation fields proved to be useful for a number of reasons. The static open fields provide a basic verification and help detect errors or deviations (either aSi‐ or linac‐related) in the practical implementation of the standardized configuration package. Possible errors, such as the selection of the wrong energy during the import in the Eclipse beam data configuration or the selection of the wrong 2D profile correction during the imager calibration, are readily intercepted. In one case, saturation of the aS500 was observed for 6 MV, 600 MU/min dose rate for the largest field sizes at SID 100 cm. This panel was subsequently excluded from the testing until the saturation issue could be solved. From the static open fields as shown in Fig. 5, it can also clearly be observed that the aSi dose measured beneath the main collimators is not well reproduced by the PDIP algorithm. All further gamma evaluation calculations are, therefore, restricted to within the collimator opening. The linearity tests reveal no new data other than the results presented above, but serve to raise the user's awareness regarding the nonlinear behavior in the case of extremely short or — to a lesser degree — very long image acquisition times.

Variations observed in the agreement between predicted and measured artificial dynamic IMRT fields could be brought back to a number of parameters.

**Figure 6:**
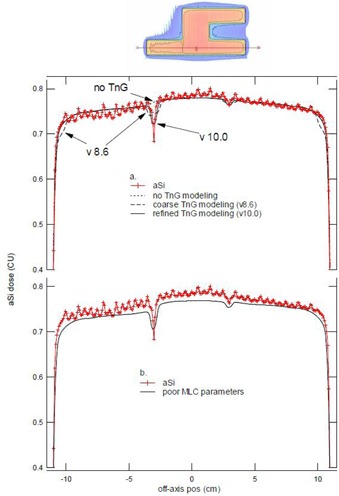
Calculated and measured profiles extracted from the artificial dynamic Chair IMRT pattern in a 12×24 collimator opening (collimator rotation set to 90°) illustrate the impact of the calculated fluence on the accuracy of the portal dosimetry results. Connected symbols (+) represent the measurement, while lines represent predictions with the standardized PDIP configuration but with (a) subsequent versions of the Eclipse actual fluence calculation software and (b) suboptimal MLC parameter modeling.

Firstly, depending on the Eclipse version used in the different departments, variations in the actual fluence calculations were reflected in the predicted image. One clearly noticeable example was the evolution of the tongue‐and‐groove modeling in the dose calculation model. The impact of this is illustrated in Fig. 6(a), using the dynamic Chair artificial IMRT pattern^(27)^ in a 12×24 collimator opening as an example. Whereas the earlier versions did not include any tongue‐and‐groove modeling whatsoever, a first and rather coarse model was introduced in version 8.6 of the leaf motion calculator (LMC), spreading the effect over the whole width of the leaf rather than introducing a small shift in the penumbra. This gave rise to considerable oversmoothening of the narrow dose dips that are characteristic for the tongue‐and‐groove effect and showed stepwise underestimation of the dose at bordering leafs (indicated by the arrows for v8.6 in Fig. 6(a)). The resolution of the tongue‐and‐groove effect was considerably improved in the more recent versions (v10.0), especially in the case of RapidArc fluence calculations.^(24)^ Although the modeling of the tongue‐and‐groove effect is not the subject of this study, it is important to note that it affects the precision with which the image predictions are reproduced by the acquired image.

Poor modeling of the user definable MLC parameters (transmission and dosimetric leaf separation) also has an impact on the accuracy with which the actual fluence is calculated and, therefore, affects the overall outcome of the portal dosimetry validation. An example of such a case is given in Fig. 6(b). It can be seen that inferior agreement is obtained when using values of 1.3 mm for the dosimetric leaf separation and 1.5% for the overall leaf transmission instead of the more favorable values used in Fig. 6(a) (1.6 mm and 1.9%, respectively).

Although the RapidArc PDIP validation plans yield good overall pass rates, a systematic deviation of+1% to 2% could be seen in the measured images when looking in more detail at the line profiles. The underlying cause for this deviation is the slightly nonlinear behavior of the portal dose acquisition. Although the RapidArc treatments were delivered with typically 300 MU at a nominal dose rate of 600 MU/min, the total acquisition time is the time required for the whole arc delivery and is typically in the order of 1.3 minutes for an arc at maximum gantry speed. Such a treatment time corresponds to the delivery of ~800MU for an open field at 600 MU/min. The observed systematic deviation is, therefore, in very good agreement with the results found in the linearity testing. The imager was calibrated to yield 1 CU for 100 MU. As most of the IMRT fields are delivered with similar MU — sometimes less, sometimes more, the effect of the nonlinearity appears to be random and is too small to stick out in clinical routine when using 3%,3 mm (or even 2%,2 mm) acceptance criteria. For RapidArc treatments, the deviation is still relatively small but quite systematic due to the near‐constant treatment delivery times. When performing the imager absolute dose calibration with 800 MU at 600 MU/min and setting the corresponding dose to 8 CU (instead of defining 1 CU for 100 MU), the systematic deviation for RapidArc portal dose acquisitions disappears.

**Figure 7:**
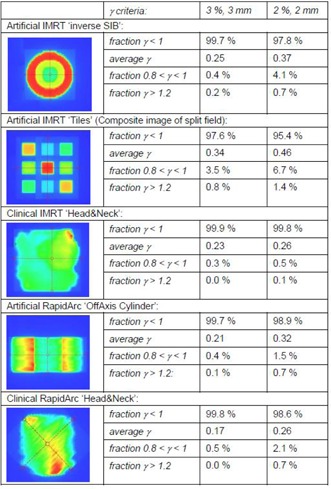
Examples of the PDIP dynamic MLC test package with the left column showing the (predicted) images. Gamma evaluation results are given for 3%,3 mm and 2%,2 mm criteria, showing the total percentage of points that passed (i.e., gamma index or ‘fraction γ<1’), the average gamma value, the fraction of points that only just passed (‘fraction0.8<γ<1’), and those that failed by a more considerable amount (‘fractionγ>1.2’). All calculations were restricted to the data points within the collimator opening.

**Figure 8:**
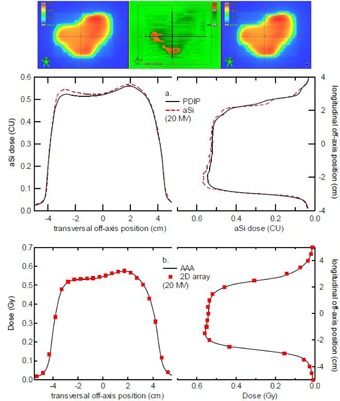
Portal dose deviations observed for the high photon energies. (a) Example of typical deviation, occasionally observed for the highest photon energies (20 MV) for small, not too modulated IMRT fields. The dose in the predicted image (left image and solid line profile) is more scattered out than the one in the acquired image (right image and dashed line profile). The black lines in the central gamma evaluation (3%,3 mm) image indicate the positions of the extracted line profiles. (b) Phantom measurements (at 5 cm depth) of the same field show good agreement between calculated (AAA) (solid lines) and measured dose (2D ion chamber array) (square symbols).

Apart from the above‐mentioned possible deviations, all acquired images easily pass the commonly used gamma evaluation criteria (pass rate of 95% at 3%,3 mm within the collimator opening) and most even pass the more strict criteria (98.5% at 3%,3 mm within the collimator opening) as proposed by Vinall et al.^(15)^ for portal dose evaluation. Figure 7 illustrates this through a number of test fields (6 MV) and the corresponding gamma evaluation outcome as calculated within the Varian portal dosimetry software. To better quantify the level of agreement, we have calculated gamma evaluations for both 3%,3 mm and 2%,2 mm criteria. In addition, for both sets of criteria, apart from the commonly reported gamma index (i.e., the fraction of the points for which γ<1), we have also listed the average gamma value, the fraction of points that only just passed (‘fraction0.8<γ<1’) and those that failed by a more considerable amount (‘fractionγ>1.2’). Only the artificial IMRT ‘Tiles' pattern results in a pass rate of 97.6%, not meeting the more stringent 98.5% criterion. The fraction of acceptable points with a elevated gamma values (0.8<γ<1) is also larger than for the other fields (3.5% instead of the more commonly observed values below 1%). The ‘Tiles' pattern is delivered through the use of two subsequent carriage positions with individual positions of the X collimators each. As a result of this, a fraction of the composite dose is inevitably measured and calculated beneath the main collimators. Keeping the above mentioned limitations of the PDIP single pencil beam algorithm beneath the main collimator in mind, a decrease in the gamma index is therefore to be expected. Even so, the 95% pass rate is still achieved (even for 2%,2 mm criteria). However, for the highest energy beams (20 MV) inferior results are sometimes observed for relatively small and moderately modulated fields. IMRT or RapidArc simple prostate treatments with 20 MV provide very typical examples, as shown in Fig. 8. The 3%,3 mm gamma evaluation shows a 95.3% gamma index and a 9.9% elevated gamma (0.8<γ<1) fraction. Using 2%,2 mm criteria, only 89.3% of the in‐field points pass (and 15.1% have an elevated gamma value). These fields were carefully cross‐checked by other methods. Validation by means of the EpiQA (EPIdos, Bratislava, Slovakia) software revealed similar deviations between the aSi measurement and the AAA calculation (not shown but very similar to the data displayed in Fig. 8(a)). However, 2D ion chamber array measurements (PTW Seven29, Freiburg, Germany) in solid water for IMRT fields (measured at isocenter at 5 cm deep) or in the OCTAVIUS phantom (PTW) for RapidArc fields, showed near‐perfect agreement with the AAA dose calculations, as shown in Fig. 8(b), indicating that both the delivery and the actual fluence calculation in the TPS are correct. It can therefore be concluded that both the EpiQA dose to water reconstruction and the PDIP algorithm fail to accurately describe the behavior of the aSi dose distribution for such small high‐energy fields. They both overestimate the outscattering of the dose, resulting in an overly smoothened calculated dose distribution. The exact cause of this small‐field deviation remains unknown to date and will require further investigation. Similar tendencies can occasionally be observed for the 18 MV or 15 MV beams, but are much smaller than for the 20 MV treatment beam and do not often give rise to failed gamma evaluations.

## III. DISCUSSION

The Varian aSi panels tested during this study show nearly identical dosimetric characteristics for all tested photon energies, providing justification for the development and use of a universal, optimized portal dose prediction configuration dataset. While the current portal dose configuration is rather time consuming and error prone, the standardized dataset reduces the entire portal dosimetry configuration time to a mere 5 minutes in the Eclipse beam configuration application and another 5 minutes on the Clinac. Additionally, most importantly, it includes previously developed workarounds to the main issues encountered within the current Varian portal dosimetry release (i.e., the suboptimal profile correction and backscatter of the retractable arm). Although the chosen backscatter correction approach is a simplified one compared to more accurate, field size‐dependent algorithms that have been proposed in the literature,^(23)^ it presents the major advantage that it can be integrated in the existing portal dosimetry solution without the need for any additional image transfer and processing, thus preserving the solution's high efficiency in clinical routine. (The strong spectral dependence of the imager panel^28^, ^29^, ^30^ is not addressed as its effect is minimal provided the imager panel is always used in its dosimetric calibration position.) As a third advantage, the use of the preconfigured data makes the PDIP configuration independent of possible flaws in the actual fluence calculation of the pyramid‐shaped fluence used during the traditional PDIP configuration process. These flaws can be either user dependent (e.g., through the use of suboptimal MLC parameters) or Eclipse version dependent (e.g., through changes in the fluence calculation algorithm). Both cases have been known to occur, as illustrated through the line profiles in the ‘Chair’ portal dose images in Fig. 6.

When opting for the preconfigured dataset, it is advisable to also run a set of validation fields similar to the ones reported in this study. These validation fields will not only check the correct implementation, they also serve to illustrate the accuracy that can be obtained with it, as well as the limitations that still exist. Provided the MLC parameters have been adequately defined, gamma pass rates of 95% of the in‐field points meeting 3%,3 mm criteria should be easily obtained. Most fields will even go beyond a 98.5% pass rate. In routine use, we therefore request a pass rate of 97% at 3%,3 mm within the collimator opening and a warning when the average gamma value exceeds 0.5 to help detect systematic deviations in the absolute dose level. The main limitations that exist are the inferior results for relatively small, moderately modulated 20 MV fields and the slightly nonlinear behavior of the integral dose acquisition as a function of acquisition time. When performing the absolute dosimetric imager calibration by means of 100 MU, the nonlinearity will only have a very small impact (< 1%) on IMRT field verification as acquisition times scale with MU and the latter are mostly in the range of 50–250 MU/field. RapidArc dosimetric images require a considerably longer acquisition time and lead to an overestimate of the overall dose level by 1%‐2%. As it is an inherent limitation of the current aSi integrated acquisition mode, it affects other commercial EPID dosimetry solutions using this imaging mode by an equal amount. Although it needs to be dealt with through a software modification by the vendor, current deviations are not large and can easily be corrected for by slightly renormalizing the acquired image or by increasing the MU used during the absolute calibration procedure to reduce the relative contribution of the missing last (partial) frame to the total dose. Although no correction for the imager sag could be applied, in agreement with Bailey et al.^(17)^ we found it not significant enough to have a considerable impact on our study. Whereas Rowshanfarzard et al.^(31)^ reported improvements in the 3%,3 mm gamma index from 85.4% to 94.1%, most of the RapidArc fields in our study already show gamma indices beyond 98.5%.

Finally, although we have taken advantage of the experimental confirmation that all aSi panels behave in a nearly identical way to obtain standardized datasets for the Varian portal dosimetry solution, the argumentation would hold equally well for other portal dosimetry applications, be it pretreatment QA or transit dosimetry (as long as the treatment machines are also well matched or differences between treatment machines can be taken into account). One of the reasons why so little of the published material has made it into widespread clinical use is because of the difficulties encountered in the data acquisition and model configuration/implementation. Standardized datasets could considerably help overcome these obstacles.

## IV. CONCLUSIONS

After having shown that all Varian aSi panels display very similar dosimetric behavior, we have developed a universal set of preconfigured PDIP datasets for the Varian portal dosimetry solution. As these preconfigured data include improved 2D profile correction files that also take the backscatter of the Exact arm into account (albeit in a simplified, field‐size independent way), they deal with the most annoying flaws in the current commercial solution. Whereas the making of an optimized PDIP configuration requires cumbersome data processing beyond the possibilities of most radiotherapy departments, importing standardized datasets is fast and easy, facilitating the transfer of optimized solutions into clinical routine. A simple set of validation fields should then suffice to assess the accuracy of the implementation on site.

## ACKNOWLEDGMENTS

The authors wish to thank their numerous colleagues for allowing them to use the data acquired on their aSi imager panels. They also wish to thank the Varian iLab team for their enthusiastic support and feedback during the development of the standardized configuration packages.

## References

[1] vanElmpt W , McDermott L , Nijsten S , Wendling M , Lambin P , Mijnheer B . A literature review of electronic portal imaging for radiotherapy dosimetry. Radiother Oncol. 2008;88(3):289–309. 18706727 10.1016/j.radonc.2008.07.008

[2] Antonuk L , El‐Mohri Y , Huang W , et al. Initial performance evaluation of an indirect‐detection, active matrix flat‐panel imaginr (AMFPI) prototype for megavoltage imaging. Int J Radiat Oncol Biol Phys. 1998;42(2):437–54. 9788427 10.1016/s0360-3016(98)00210-7

[3] McDermott L , Louwe R , Sonke J , van Herk M , Mijnheer B . Dose‐response and ghosting effects of an amorphous silicon electronic portal imaging device. Med Phys. 2004;31(2):285–95. 15000614 10.1118/1.1637969

[4] Winkler P , Hefner A and Georg D . Dose response characteristics of an amorphous silicon EPID. Med Phys. 2005;32(10):3095–105. 16279061 10.1118/1.2040711

[5] Greer P and Popescu C . Dosimetric properties of an amorphous silicon electronic portal imaging device for verification of dynamic intensity modulated radiation therapy. Med Phys. 2003;30(7):1618–27. 12906179 10.1118/1.1582469

[6] Van Esch A , Depuydt T , Huyskens D . The use of an aSi‐based EPID for routine absolute dosimtric pre‐treatment verification of dynamic IMRT fields. Radiother Oncol. 2004;71(2):223–34. 15110457 10.1016/j.radonc.2004.02.018

[7] McCurdy BM , Luchka K , Pistorius S . Dosimetric investigation and portal dose image prediction using an amorphous silicon electronic portal imaging device. Med Phys. 2001; 28(6):911–24. 11439488 10.1118/1.1374244

[8] Nicolini G , Fogliata A , Vanetti E , Clivio A , Cozzi L . GLAaS: an absolute dose calibration algorithm for an amorphous silicon portal imager. Applications to IMRT verifications. Med Phys. 2006;33(8):2839–51. 16964860 10.1118/1.2218314

[9] Gustafsson H , Vial P , Kuncic Z , Baldock C , Denham JW , Greer PB . Direct dose to water dosimetry for pretreatment IMRT verification using a modified EPID. Med Phys. 2011;38(11):6257–64. 22047391 10.1118/1.3656946

[10] Vial P , Greer P , Oliver L , Baldock C . Initial evaluation of a commercial EPID modified to a direct‐detection configuration for radiotherapy dosimetry. Med Phys. 2008;35(10):4362–74. 18975682 10.1118/1.2975156

[11] Sabet M , Menk F , Greer P . Evaluation of an a‐Si EPID in direct detection configuration as a water‐equivalent dosimeter for transit dosimetry. Med Phys. 2010;37(4):1459–67. 20443467 10.1118/1.3327456

[12] Greer P , Cadman P , Lee C , Bzdusek K . An energy fluence‐convolution model for amorphous silicon EPID dose prediction. Med Phys. 2009;36(2):547–55. 19291994 10.1118/1.3058481

[13] Parent L , Seco J , Evans P , Fielding A , Dance D . Monte‐Carlo modelling of aSi EPID response: the effect of spectral variations with field size and position. Med Phys. 2006;33(12):4527–40. 17278804 10.1118/1.2369465

[14] Siebers J , Kim J , Ko L , Keall P , Mohan R . Monte Carlo computation of dosimetric amorphous silicon electronic portal images. Med Phys. 2004;31(7):2135–46. 15305468 10.1118/1.1764392

[15] Vinall A , Williams A , Currie V , Van Esch A , Huyskens D . Practical guidelines for routine intensity‐modulated radiotherapy verification: pre‐treatment verification with portal dosimetry and treatment verification with in vivo dosimetry. Br J Radiol. 2010;83(995):949–57. 20965905 10.1259/bjr/31573847 PMC3473728

[16] Vial P , Greer PB , Hunt P , Oliver L , Baldock C . The impact of MLC transmitted radiation on EPID dosimetry for dynamic MLC beams. Med Phys. 2008;35(4):1267–77. 18491519 10.1118/1.2885368

[17] Bailey DW , Kumaraswamy L , Bakhtiari M , Malhotra HK , Podgorsak MB . EPID dosimetry for pretreatment quality assurance with two commercial systems. J Appl Clin Med Phys. 2012;13(4):82–99. 10.1120/jacmp.v13i4.3736 PMC5716510 22766944

[18] Ko L , Kim J , Siebers J . Investigation of the optimal backscatter for an aSi electronic portal imaging device. Phys Med Biol. 2004;49(9):1723–38. 15152927 10.1088/0031-9155/49/9/010

[19] Moore J and Siebers J . Verification of the optimal backscatter for an aSi electronic portal imaging device. Phys Med Biol. 2005;50(10):2341–50. 15876671 10.1088/0031-9155/50/10/011

[20] Rowshanfarzad P , Sabet M , O'Connor D , Greer P . Reduction of the effect of non‐uniform backscatter from an E‐type support arm of a Varian a‐Si EPID used for dosimetry. Phys Med Biol. 2010;55(22):6617–32. 20962364 10.1088/0031-9155/55/22/003

[21] Rowshanfarzad P , McCurdy B , Sabet M , Lee C , O'Connor D , Greer P . Measurement and modelling of the effect of support arm backscatter on dosimetry with a Varian EPID. Med Phys. 2010;37(5):2269–78. 20527561 10.1118/1.3369445

[22] Greer PB , Cadman P , Lee C , Bzudsek K . An energy fluence convolution model for amorphous silicon EPID dose prediction. Med Phys. 2009;36(2):547–55. 19291994 10.1118/1.3058481

[23] Berry S , Polvorosa C , Wuu C . A field size specific backscatter correction algortihm for accurate EPID dosimetry. Med Phys. 2010;37(6):2425–34. 20632552 10.1118/1.3400043

[24] Van Esch A , Huyskens DP , Behrens CF , et al. Implementing RapidArc into clinical routine: a comprehensive program from machine QA to TPS validation and patient QA. Med Phys. 2011;38(9):5146–66. 21978060 10.1118/1.3622672

[25] Sjöstrom D , Bjelkengren U , Ottosson V , Behrens CF . A beam‐matching concept for medical linear accelerators. Acta Oncol. 2009;48(2):192–200. 18752079 10.1080/02841860802258794

[26] McCurdy BMC and Greer P . Dosimetric properties of an amorphous‐silicon EPID used in continuous acquisition mode for application to dynamic and arc IMRT. Med Phys. 2009;36(7):3028–39. 19673202 10.1118/1.3148822

[27] Van Esch A , Bohsung J , Sorvari P , et al. Acceptance tests and quality control (QC) procedures for the clinical implementation of intensity modulated radiotherapy (IMRT) using inverse planning and the sliding window technique: experience of five radiotherapy departments. Radiother Ocol. 2002;65(1):53–70. 10.1016/s0167-8140(02)00174-3 12413675

[28] Greer P . Correction of pixel sensitivity variations and off‐axis response for amorphous silicon EPID dosimetry. Med Phys. 2005;32(12):3558–68. 16475754 10.1118/1.2128498

[29] Greer P . Off‐axis dose response characteristics of an amorphous silicon electronic portal imaging device. Med Phys. 2007;34(10):3815–24. 17985627 10.1118/1.2779944

[30] Partridge M , Hesse B , Müller L . A performance comparison of direct‐ and indirect‐detection flat‐panel imagers. Nucl Instrum Methods Phys Res. A. 2002;484(1–3):351–63.

[31] Rowshanfarzad P , Sabet M , O'Connor DJ , McCowan PM , McCurdy BMC , Greer P . Detection and correction for EPID and gantry sag during arc delivery using cine EPID imaging. Med Phys. 2012;39(2):623–37. 22320771 10.1118/1.3673958

